# Bulky Ligand-Induced Hindrance in Photocatalytic CO_2_ Reduction over Various Tris(bipyridine)cobalt(II) Chloride Complexes

**DOI:** 10.3390/molecules30122573

**Published:** 2025-06-13

**Authors:** Jinliang Lin, Rongying Liao, Li Li, Shuli Yao, Shengkai Li, Yun Zheng, Fei Fei

**Affiliations:** 1 School of Intelligent Manufacturing and Materials Engineering, Gannan University of Science and Technology, Ganzhou 341000, China; liaorongying@gnust.edu.cn (R.L.); 9320080302@gnust.edu.cn (L.L.); 9320220065@gnust.edu.cn (S.Y.); 9320230036@gnust.edu.cn (S.L.); 2 Fujian Provincial Key Laboratory of Biomass Low-Carbon Conversion, Huaqiao University, Xiamen 361021, China; zheng-yun@hqu.edu.cn; 3 Department of Chemical and Engineering, Zunyi Normal University, Zunyi 563000, China; feifei90092@163.com

**Keywords:** CO_2_ reduction, photocatalysis, ligand effect, electrochemistry

## Abstract

Photocatalytic CO_2_ conversion is one of the ideal approaches to address both topics of solar energy shortage and carbon neutrality. Cobalt(II) centers coordinated with bipyridines have been designed and evaluated as catalysts for CO_2_ conversion under light irradiation. Herein, we report a series of pyridine-based cobalt complexes with alkyl substituents as molecular photocatalysts, aiming to elucidate the effects of alkyl type and substitution position on catalytic performance through spectroscopic and electrochemical measurements. The substitution of the hydrogen at 4,4′-positions on the bipyridine ring with a methyl group, a tert-butyl group, and a nonyl group led to a decrease in the conversion rate of CO_2_ by 13.2%, 29.6%, and 98%, respectively. The methyl substituents at the 5, 5′-positions of the bipyridine ring resulted in a 71.1% decrease in the CO_2_ conversion rate. The usage of either 6, 6′-Me_2_-2,2′-bipy, 2,4-bipy, or 3,3′-bipy resulted in no detectable activity for CO_2_ conversion in the current system. Both photo- and electrochemical analyses have been employed to reveal the relationship between changing ligands and photocatalytic performance on the molecular scale. These results demonstrate that bulky ligands significantly hinder CO_2_ reduction by cobalt complexes due to steric interference with coordination and active-site accessibility. This study demonstrates that the substituent effect of ligands on photocatalytic reactions for CO_2_ conversion provides valuable insight into a deeper understanding of molecular catalysis.

## 1. Introduction

The enormous consumption of fossil fuels has led to a continuously increasing concentration of carbon dioxide (CO_2_) in the atmosphere, resulting in climate change and other environmental issues [1]. Therefore, it is urgent to identify clean and renewable energy sources to reduce atmospheric CO_2_ equivalents (CO_2_e) and meet growing energy demands. Photocatalytic CO_2_ conversion into value-added chemical products is widely regarded as a promising approach to producing renewable energy and alleviating global climate change [2]. Over the past five decades, significant progress has been made in CO_2_ activation and transformation through a variety of approaches, including theoretical simulations, spectroscopic analyses, experimental investigations, and mechanistic studies [3,4,5]. Despite ongoing research endeavors dedicated to the photocatalytic conversion of CO_2_, the attainment of commercially viable CO_2_ fixation remains challenging.

For the purpose of high photocatalytic efficiency, numerous studies have focused on the development of materials for CO_2_ conversion. These materials, used as catalysts, can generally be classified into two types: semiconductors and molecular complexes along with their derivatives [1,4,5,6]. In recent years, different types of new materials have been explored as efficient catalysts for CO_2_ photoreduction. For instance, metal–organic frameworks (MOFs) and/or covalent organic frameworks (COFs), which possess ultrahigh surface areas (>3000 m^2^/g) and tailorable active sites, show great promise for CO_2_ photoreduction [7,8]. NH_2_-UiO-66(Zr) obtained at 473 K achieves HCOOH production as high as 129.8 μmol·g^−1^·h^−1^ [9]. Visible-light-responsive (λ > 420 nm) materials such as g-C_3_N_4_/Pt/BiVO_4_ nanocomposites exhibit 72% quantum efficiency for CH_4_ generation [10]. MXene/MOF composites (e.g., Ti_3_C_2_T_x_/ZIF-67) exhibit exceptional conductivity, enabling fivefold faster charge transfer than pure MOFs, which results in CO production at 62.7 μmol·g^−1^·h^−1^ [11]. Beyond the UV–vis light irradiation, another breakthrough involves hydroxyl-bonded Ru on metallic TiN surfaces, which also shows promising catalytic properties for CO_2_ reduction with H_2_O under infrared light [5]. Among these presented candidates, the first-generation materials for photocatalytic CO_2_ reduction, namely metal-complex molecular catalysts, should be highlighted because of their merits of intensive visible light absorption, high quantum efficiency, and good product selectivity [12]. The metal centers coordinated with functional ligands and exposed to the reaction medium provide sufficient active sites, which greatly reduce the kinetic barriers during the catalytic reactions [13]. In particular, the nature of the coordinating ligands could alter the electronic properties of the metal centers and then affect the catalytic performance. Therefore, a suitable ligand is crucial to achieve excellent catalytic properties for molecular catalysts.

The initial use of Ru(bipy)_3_Cl_3_ and Co(bipy)_3_Cl_2_ as photocatalysts opened a new horizon for molecular catalysts in the field of solar energy conversion and CO_2_ reduction [14]. Subsequent research showed that altering the kinds of metal centers could affect the properties of metal bipyridine complexes (referred as M-bipy), such as interaction force, geometry, charge distribution, and subsequent photocatalytic performance. Therefore, various metal complexes have been successfully prepared and then employed as catalytic materials in CO_2_ reduction [15]. The coordination of a molecular nickelous quaterpyridine (Ni-qpy) exhibits selective catalytic activity for CO_2_ reduction in water [16,17]. In addition, replacement of the primary ligands with secondary ones or modifications of the ligand scaffold may also produce similar results to the regulation of metal centers, including influencing electronic and steric properties and, thus, affecting catalytic performance [12]. For example, the introduction of proton donors to both Re(bipy)(CO)_3_Cl and Mn(bipy)(CO)_3_Br complexes has been reported to help stabilize the CO_2_ adduct during catalysis and increase catalytic activity via hydrogen bonding interactions [18]. Moreover, terpyridine (tpy) and quaterpyridine (qpy) complexes have also been extensively studied for both photocatalytic water splitting and CO_2_ reduction [19]. In fact, there are numerous studies introducing various functional groups onto bipyridines to regulate their electron donating and accepting abilities [20].

As we know, polypyridines are a typical class of complexes for excited electron transfer and molecular activation when they coordinate with various metal ions during photocatalytic CO_2_ conversion. Their excellent performance is mainly attributed to their high oxidative stability and π-accepting ability, which can maintain the stability of metal centers in both high and low oxidation states. In particular, relatively small overpotentials are beneficial for the occurrence of CO_2_ reduction via a low-valent active intermediate. The most commonly used pyridines are those comprising one to four pyridine rings, which help minimize steric hindrance during the entire catalytic process. Moreover, the molecular structures of these small complexes can be easily tuned by adding various substituents to the M-bipy framework. The semiconductor–complex (CdS+M(bipy)_3_Cl_2_, M=Co, Ni) suspension catalytic system provides an excellent research platform, taking advantage of both operational simplicity and high efficiency [21,22].

Herein, the relationship between different ligands and the photocatalytic CO_2_ performance based on Co-bipy complexes and their derivatives has been demonstrated. The effects of the 4,4′-substituents (OCH_3_, CH_3_tBu, H, CN, CF_3_) and relevant ligands on Re-bipy-based catalysis have been reported previously [17]. In this study, various types of alkyl substituent groups and coordination positions were systematically investigated. The catalytic performance of CO_2_ conversion was found to be closely related to the 2,2′-bipyridine complexes, with bulky alkyl side groups significantly hindering the CO_2_ reduction by the cobalt complexes. That is, substitution of the hydrogen at position four on the bipyridine ring with a methyl group, a tert-butyl group, and a nonyl group led to a decrease in the conversion rate of CO_2_ by 13.2%, 29.6%, and 98%, respectively. When the hydrogen at position five on the bipyridine ring was replaced by methyl, the corresponding reduction in the conversion rate was 71.1%. The usage of either 6,6′-Me_2_-2,2′-bipy, 2,4-bipy, or 3,3-bipy resulted in no detectable activity for CO_2_ conversion in the current system. Photochemical, electrochemical, and spectroscopic techniques have been employed to detect coordination information and transition states of the complexes used. Spectroscopic measurements confirm the in situ formation of cobalt–pyrimidine complexes. Moreover, a possible process involving photoexcited charge transfer and intermediate species transformation in various ligands is discussed.

## 2. Results and Discussion

### 2.1. Photocatalytic CO_2_ Reduction

**Figure 1:**
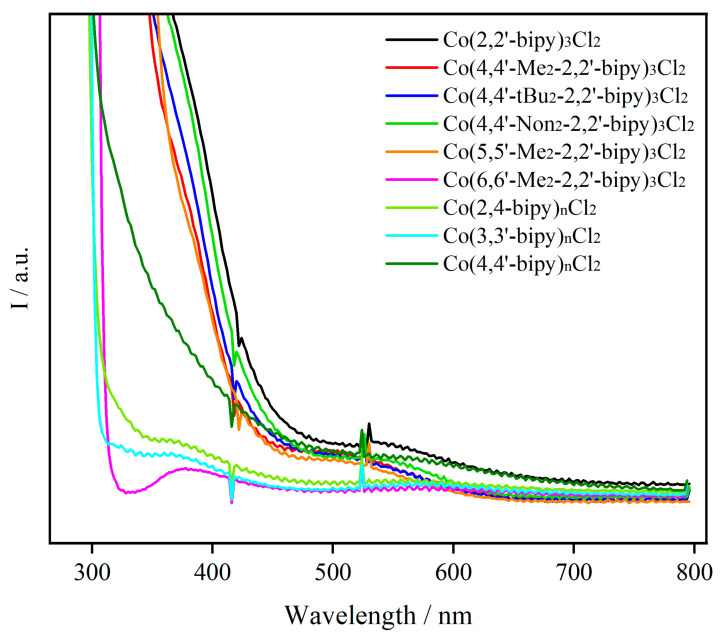
UV–vis absorption spectra of cobalt complexes with various ligands.

A series of control experiments were conducted before the tests of various ligands, and the results of photocatalytic CO_2_ reduction performance under different conditions are listed in Table 1. After 2 h of visible light irradiation (λ > 420 nm), the activity of the system without any cobalt complex is moderate, while [Co(bipy)_3_]^2+^ alone is inactive in the reaction (Entries 1 and 2). No obvious CO evolution was observed, but H_2_ production was increased when the system comprised CdS with Co^2+^ (Entry 3). This indicates that the solitary units cannot constitute an active complex. This result is consistent with the absorption spectra (Figure 1). These tests have been previously performed and were repeated here as reference samples (Table S2) [12].

Consequently, the addition of cobalt complexes, comprising cobalt centers associated with different ligands, has been observed to influence catalytic performance. The model system for photocatalytic CO_2_ reduction comprising CdS, Co(II), and 2,2′-bipy exhibited good performance; it produced 40.8 μmol of CO production and 8.6 μmol of H_2_ evolution (Entry 6, Table 1). Then, various alkyl groups substituted on the bipyridine ligands were utilized to explore their impact on catalytic behavior. From entries 6 to 9 (Group 1), it can be observed that the introduction of alkyl groups leads to a decrease in CO and H_2_ evolution, with a further elongation of the alkyl chain exacerbating this effect. Specifically, CO production decreased to 35.4 μmol (13.2%), 28.7 μmol (29.6%), and 0.8 μmol (98%), and H_2_ production to 7.5 μmol, 6.2 μmol, and 1.5 μmol for 4,4′-Me_2_-2,2′-bipy, 4,4′-tBu_2_-2,2′-bipy, and 4,4′-Non_2_-2,2′-bipy, respectively. In particular, the generation and selectivity of CO at 4,4′-Non_2_-2,2′-bipy are much lower than those of the other examined pyridine ligands, which is mainly due to the long nonyl chain causing severe steric hindrance and mass transfer resistance. The selectivity for CO generally follows the order of 2,2′-bipy, 4,4′-Me_2_-2,2′-bipy, 4,4′-tBu_2_-2,2′-bipy, and 4,4′-Non_2_-2,2′-bipy.

Comparing the used ligand bipy to 4,4′-Me_2_-2,2′-bipy, 5,5′-Me_2_-2,2′-bipy, and 6,6′-Me_2_-2,2′-bipy (Group 2), it is observed that they retain catalytic activity when the methyl is attached at the 4,4′- /5,5′- position (Entry 10 and 11, Table 1), while the production of CO is not detectable for 6, 6′-Me_2_-2,2′-bipy (Entry 11, Table 1). The sample of 6, 6′-Me_2_-2,2′-bipy shares the same formula weight and functional groups; therefore, the possibility of mass transfer resistance should be excluded from the photocatalytic reaction. From the structure of 6,6′-Me2-2,2′-bipy, it is evident that the nitrogen atom adjacent to the methyl and pyridine ring prevents its coordination with a single cobalt center. This aspect will be further elucidated through photo-/electrochemical measurements. To gain a more specific understanding of the steric effect on photocatalytic CO_2_ reduction, two pyridine rings connected at different positions were selected for this experiment. As listed in Entries 12~14 of Table 1 (Group 3), 3,3′-bipy and 2,4-bipy did not possess the capacity for photocatalytic CO_2_ activity, and 4,4′-bipy presented reduced activity. This can be attributed to two para-position nitrogen atoms being difficult to simultaneously coordinate with one cobalt center. The activity of Co-4,4′-bipy can be interpreted as two 4,4′-bipy ligands being equivalent to one bipy ligand, associating with a cobalt ion without steric hindrance due to the presence of two nitrogen atoms distributed on both ends of a linear 4,4′-bipy molecule. All these results indicate that the coordination capability of the ligands significantly affects the performance of photocatalytic CO_2_ reduction.

### 2.2. UV–Vis Absorption

The UV–vis absorption spectra were collected in the reaction media by directly dissolving CoCl_2_ (0.1 mmol) in a solution of MeCN/TEOA/H_2_O containing various ligands. As shown in Figure 1, the UV–vis absorption spectra obtained from the solution generally show a strong absorption band. It is evident that the formation of complexes with different ligands leads to successive spectral changes. The absorption bands located at around λ < 300 nm are attributed to metal-to-ligand charge transfer (MLCT) bands [23]. Most of the complexes showing absorption bands from approximately 315 nm to 400 nm have been experimentally attributed to coordinated aromatic rings (π–π* transition), where coordination with the cobalt ion occurs [24]. From this figure, negligible absorption is observed at the current absorption band for cobalt complexes of 2,4-bipy, 3,3′-bipy, and 6,6′-Me_2_-2,2′-bipy, while moderate absorption is observed for 4,4′-bipy. The alteration of these bands can be attributed to the interaction between the Co center and pyridine-based ligands, which exhibit lower polarity compared to MeCN. Additionally, dispersion forces should also be considered in this scenario.

In the case of the absorption peak at 495~580 nm, an electron is transferred from the electron-rich cobalt center to the electron-accepting bipyridine-based system (d→π* transition) upon excitation, accompanied by a simultaneous change in polarizability and dipole moment. This results in an excited state with a reduced dipole moment [25]. Similar to the previous absorption band, a nearly flat line is observed in the samples of cobalt complexes of 2,4-bipy, 3,3′-bipy, and 6,6′-Me_2_-2,2′-bipy. This implies incomplete coordination or dissociation of the ligand from the cobalt center. Other complexes exhibited similar absorption behavior and generated an absorption band around this wavelength throughout the entire UV–vis absorption test. The varying absorption intensity of these complexes indicates that the cobalt center tends to coordinate with bipy ligands rather than with solvent molecules. All these changes are mainly induced by variations in the chemical nature of ligand molecules, such as charge transfer between the ligand and metal, ligand-dependent aggregation, and complexation. Moreover, a distinct absorption intensity indicates that a cobalt bipyridine-based complex was formed.

### 2.3. FT-IR Analysis

**Figure 2:**
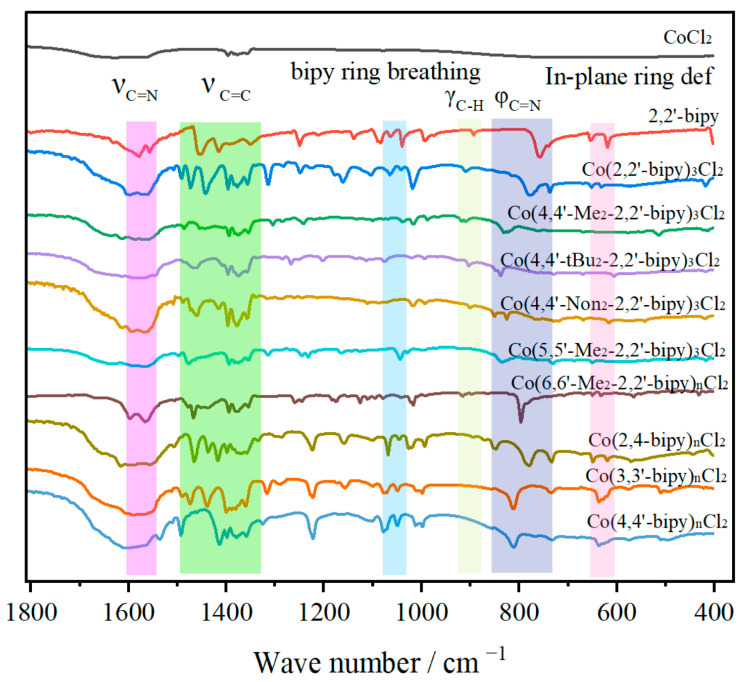
FT-IR spectra of cobalt complexes with various ligands.

As discussed above, the metal center easily coordinates with various ligands even in a turbulent reaction medium. The measurement of coordination-induced IR spectral changes has been extensively used in spectroscopic studies of ligand–metal interactions. Thus, we attempted to obtain the IR transmission characteristics of these complexes by IR spectroscopy, as shown in Figure 2. The IR spectra of some of the tested bipy ligands have already been reported, and several characteristic peaks in the region from 1000 to 1650 cm^−1^ were ascribed to the ν_C-C_ and ν_C=N_ heterocycle stretching vibrations. The absorption peak at ~1062 cm^−1^ was assigned to the ring breathing mode of the uncoordinated bipy (Figure 2) [26,27]. A relatively weak peak at 908 cm^−1^ is assigned to out-of-plane γ_C-H_ bending, which is red-shifted to 921 cm^−1^ when in contact with the cobalt center. In addition, the absorption peak at 1578 cm^−1^, associated with the ν_C=N_ stretching frequency of the free ligand, is shifted to 1550 cm^−1^ in the complex, indicating coordination of the pyridyl nitrogen to the metal ion. This shift can be attributed to the delocalization of electron density from the metal ion into the π-system of the ligand (HOMO → LUMO) [28]. Moreover, by comparing the spectra of bipy and Co-bipy, the new peak of pyridine skeletal breathing vibration at 1015 cm^−1^ is observed in these complexes. The peak at 765 cm^−1^ from φ_C=N_ torsion is red-shifted to 798 cm^−1^, and the absorption peak at 613 cm^−1^, originating from the in-plane ring deformation of free bipy, is red-shifted to 630 cm^−1^. It indicates the coordination through the pyridyl nitrogen atom. Several bands present in the region 1346~1399 cm^−1^, originating from cobalt chloride hydrate, partially overlay the absorption of ν_C-C_ on pyridine. The enhanced absorption of ν_sC-C_ at 1419 cm^−1^ and ν_asC-C_ at 1458 cm^−1^ can also be identified, which are red-shifted to 1394 cm^−1^ and 1498 cm^−1^ after the coordination [29]. Some of the coordination information was obtained by single-crystal X-ray diffraction, and the results are listed in Table S1. Meanwhile, visual color changes were observed when the ligand was mixed with cobalt species, further indicating interactions between the ligands and the metal. The absorption bands of 4,4′-Non_2_-2,2′-bipy and Co(4,4′-Non_2_-2,2′-bipy)_3_Cl_2_ at 3180 cm^−1^, 3035 cm^−1^, and 2920 cm^−1^ are ascribed to ν_N-H_, aromatic ν_C-H_, and aliphatic ν_C-H_ stretching, respectively (Figure S4). It should be mentioned that the strong absorption band of ν_O-H_ around 3600~2800 cm^−1^, originating from cobalt chloride hydrate, overlaps with the absorption of ν_N-H_ and ν_C-H_ (Figures S1–S9).

To explore further coordination information of the ligands and cobalt center, a series of bipy-based compounds with various substituents and different coordination positions were tested. Interestingly, all these tested samples exhibited different intensities at the peak of 1597 cm^−1^, thereby verifying the formation of coordination between them. This result was further supported by visual observation, as indicated by their color changes. For example, the white color of bipy turns brown after it is mixed with Co(II). Upon mixing with Co(II), the color change also occurred for the following ligands: 4,4′-Me_2_-2,2′-bipy (yellow to white), 5, 5′-Me_2_-2,2′-bipy (yellow to white), 6, 6′-Me_2_-2,2′-bipy (yellow to green), 2,4-bipy (yellow to pink), 3,3′-bipy (white to light pink), and 4,4′-bipy (yellow to pink). For Group 1, the IR spectrum of alkyl substituents on the pyridine skeleton remains similar to that of bipy, without causing the disappearance or generation of new absorption bands. However, different substitution positions of methyl groups (Group 2) result in the disappearance or shift of the peak at 1160 cm^−1^, and the peak at 1015 cm^−1^ vanishes in 5,5′-dimethyl-substituted bipy. This phenomenon is primarily attributed to steric hindrance and the inductive effect, which weaken the binding force between the ligands and the metal center. In the case of Group 3, where the distance between the two nitrogen atoms on the pyridine ring is significant, the ligands consistently function as monodentate. The color change observed in 5,5′-dimethyl-2,2′-bipy upon mixing with cobalt ions further confirms the coordination process. The details of the color changes associated with chelation are presented in Figures S1–S9.

### 2.4. Electronic Observation

**Figure 3:**
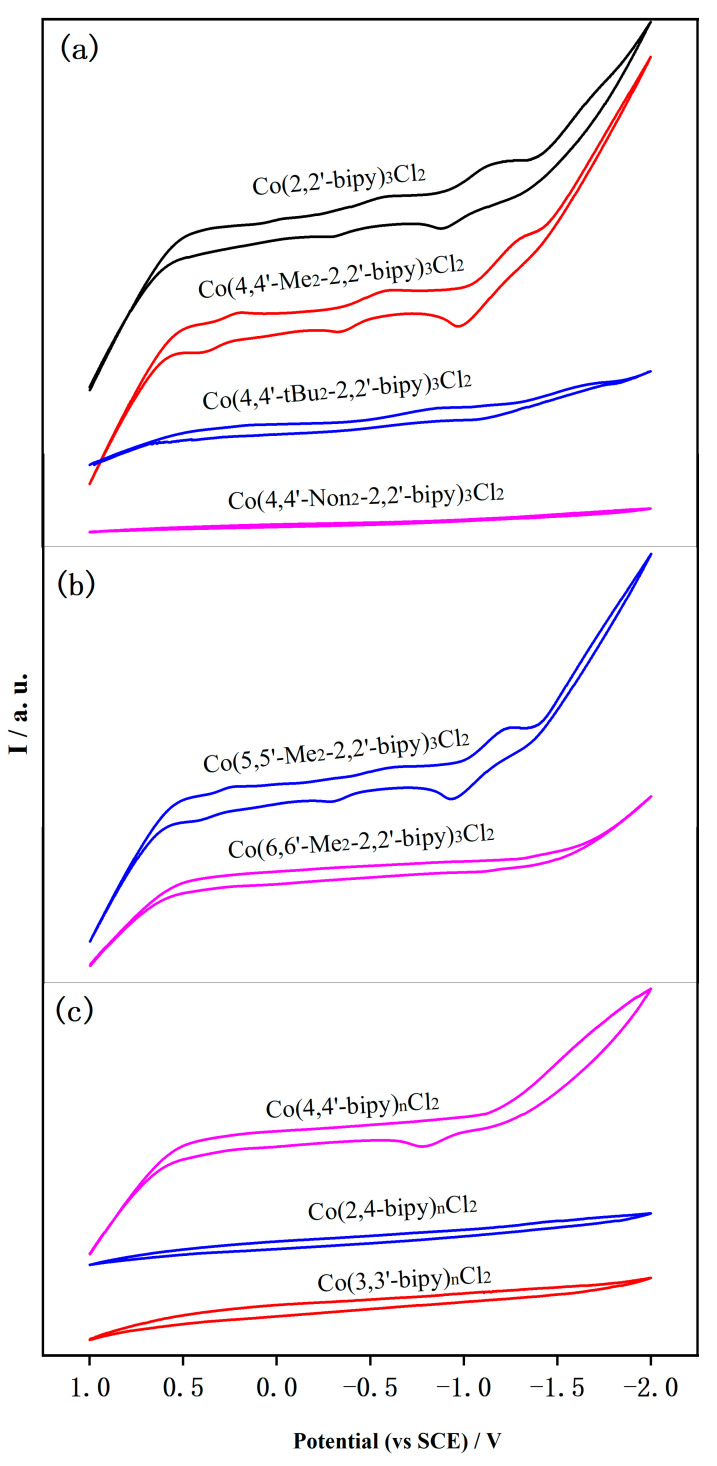
Cyclic voltammograms of the reduction waves of Co-species in the electrolytes containing various ligands after CO_2_ saturation. (**a**) various alkyl group substituted on 4, 4’-positions of pyridine rings. (**b**) different substituted positions of pyridine rings by methyl group. (**c**) different kinds of bipyridines.

The electrochemical behavior of CO_2_ reduction is then studied using cyclic voltammetry (CV). In these tests, the processes of CO_2_ reduction can be monitored indirectly, involving the initial reduction of Co^II^ complexes and their subsequent reaction with CO_2_ to form Co-CO_2_ intermediates. The resulting Co^I^ complexes are important redox-active catalysts [13]. In the tests, the reduction potential of CO_2_ was found to be close to the thermodynamic value. The obtained CV curves are shown in Figure 3 and Figures S10–S18. In the solution containing bipy, from which O_2_ has been removed by bubbling with N_2_, the observed redox potential (*E*_0_ = −0.7 V) is attributed to the conversion of the Co^II^ complex to its Co^I^ counterpart [30]. At this stage, the low valent Co intermediates are stabilized by the pyridine ligand through weak coordination [31], which promotes the activation of CO_2_ molecules via nucleophilic attack. As shown in Figure S10, another couple of redox peaks are detected (*E*_0_ = −0.4 V, and *E*_0_ = −1.2 V), possibly caused by the protonation of Co^I^ to produce Co^I^-H complex, subsequently decreasing the reduction potential [32]. The reduction peak (*E*_0_ = −0.4 V) is assigned to H_2_ evolution, as confirmed by a similar experiment performed in H_2_O [21]. In the presence of CO_2_, the corresponding peaks observed in Ar shift toward the anodic direction by approximately 500 mV, and the intensities of peaks are significantly enhanced. The markedly increased current densities indicate that the reaction systems undergo electrocatalytic CO_2_ reduction reactions with high efficiency.

As shown in Figure 3, different alkyl substituents on the pyridine ring result in changes in the CV responses in CO_2_-saturated solution. Specifically, the presence of m − ethyl groups causes a more negative shift in the cathodic potential at −1.02 V, ranging from approximately 30 to 80 mV compared to the reduction of bipy [33]. A comparable shift was previously reported for Mo-bipy, attributed to the electron donation effect of methyl substituents that elevate the LUMO (bipy) energy [34]. When the methyl group is replaced by tert-butyl, a weaker CV response is obtained, mainly due to mass transfer resistance and steric hindrance. We assume that the former factor dominates the shape, because the responses are severely weakened as the chain length further increases. Consequently, when using 4,4′-Non_2_-2,2′-bipy as a ligand, the cathodic wave at −0.4 V and −1.2 V was observed without any evidence of significant current growth associated with catalytic CO_2_ reduction. Symmetrical replacement of the hydrogen atoms in the 4,4′- and 5,5′-positions on 2,2′-bipy by methyl groups (Group 2) did not cause any obvious effect. However, a much weaker response occurred for 6,6′-Me_2_-2,2′-bipy ligands (Figure 3b), which is consistent with the measurement of the UV–vis spectrum. These observations suggest that the migration of the methyl groups on bipy has only a limited electronic, even negative, effect on the LUMO of the parent compounds and the stability of the carbonate radical anion in solution. As for Group 3, concerning the linking positions of the two pyridine rings, an almost flat CV line has been observed in the reaction medium containing the 3,3′-bipy and 2,4-bipy ligands, while a moderate response was observed in the sample with the addition of 4,4′-bipy ligand. Notably, all of these observations are in good agreement with the UV–vis spectra. From the results of UV–vis and CV measurements, it can be concluded that variations in electrochemical and photochemical properties mainly result from differences in the binding strength between the metal center and the ligand.

### 2.5. Mechanism Discussion

The relationship between the results of CO_2_ reduction evaluation and different substituents on the ligand in such a system has been revealed by photo-/electrochemical characterizations [35]. As reported in previous works, the photocatalytic CO_2_ conversion mediated by cobalt complexes can generally be described as follows: semiconductor (CdS) is excited upon light irradiation and then releases electrons [36,37]. Subsequently, the cobalt center accepts electrons from the surface of the photocatalyst, leading to the formation of the active intermediate of the Co^I^ complex. At this stage, the combination of Co^I^ intermediate with protons likely leads to H_2_ evolution and the formation of Co^III^-hydride [35]. Also, the cobalt ion allows a strong interaction with CO_2_ to form a metal carbonate. After the formation of metal carbonate, the negative charge on the oxygen atom of CO_2_ increases due to back-donation from an occupied metal orbital to an unoccupied π* orbital of CO_2_. Meanwhile, the protonation is promoted, thus providing an oxygen atom from activated CO_2_ molecules by a proton to yield the reduction product CO [38]. During the processes, the properties of cobalt complexes associated with different ligands critically affect the generation and stability of active species in the liquid medium. Considering the interaction between the cobalt ion and ligands, the process of charge transfer between the metal ion and coordinated CO_2_ is inevitably influenced by the nature of organic compounds. To elucidate this more clearly, these photographs of reaction media containing different ligands have been taken after 30 min of visible light irradiation (Figure S9). From these photos, a dark green color means the formation of Co(I) species [36,38]. However, some of the reaction media remained yellow, coincidentally, the CO_2_ conversion of these samples are always low or negligible. Compared to the bare cobalt ion without ligand coordination, the CO evolution mediated by cobalt–bipy was consistently enhanced. This result indicates that bipy is a good π-acceptor ligand capable of stabilizing metals in low oxidation states. CO_2_ reduction can occur at relatively low overpotentials via a low-valent active intermediate. This provides an opportunity to incorporate an additional small ligand, with CO_2_ being a suitable candidate.

Additionally, most of the methyl-substituted bipyridines retain the capacity for photocatalytic CO_2_ reduction. In Group 1, the tertiary butyl- and n-octyl-substituted bipy serve as a control group to the original bipy. Large substituents may prevent them from being adequately coordinated with the metal center, and also act as steric barriers to CO_2_ binding. In particular, extremely low catalytic activity was observed for the Co(4,4′-Non_2_-2,2′-bipy) complex, mainly attributed to both mass transfer resistance and steric hindrance effect. Regarding substituent position, steric effects may also impede complex formation and/or CO_2_ activation. It has been clarified in the samples of Group 2 that both positions next to the nitrogen atom on 6,6′-dimethyl-2,2′-bipy are occupied by an adjacent methyl and another pyridine ring, respectively. This partially hinders the nitrogen atom from combining with the metal center to form active species. As observed from the photos of Figure S6, it indeed possesses the capacity to chelate with the cobalt center. However, the mechanism of CO_2_ reduction mediated by Co(4,4′-Non_2_-2,2′-bipy) is still ambiguous and should be further investigated via advanced strategies. Certainly, these experimental data indicate that substitution at the 4,4′- or 5,5′- positions has a negligible effect on the coordination behavior and subsequent catalysis. In fact, both UV–vis spectra and CV measurements support the above interpretation. The relationship between photocatalytic performance and steric effect was further determined by changing the coordination position of two pyridine rings. A comparison of photocatalytic CO_2_ reduction was carried out among 3,3′ -bipy, 4,4′-bipy, and 2,4-bipy (Group 3), and Co-2,2′-bipy is also used as a control sample. Only a trace amount of CO was detected in Co-4,4′-bipy, while neither Co-3,3′-bipy nor Co-2,4-bipy could produce CO under the same condition. The UV–vis spectra shed light on the crucial step that the ligands are partially chelated with the cobalt center. Each bipyridine molecule exhibits a significant distance between the two “N” atoms, enabling chelation with a single cobalt center. Remarkably, Co-4,4′-bipyridine demonstrates catalytic capacity for the conversion of CO_2_ to CO, suggesting a similarity in electron distribution shared by cobalt. We speculate that its similar electron distribution to Co-bipy is attributed to the two para-positioned methyl groups, which simultaneously chelate with a single cobalt ion to balance the polarity of each component. However, 3,3′-bipyridine and 2,4-bipyridine are unlikely to form a suitable structure and construct efficient electron delivery pathway during CO_2_ activation and conversion.

## 3. Materials and Methods

### 3.1. Materials

All chemicals were purchased from commercial suppliers and used without further purification: Cadmium sulfide (CdS, Aladdin Holdings Group Co., Ltd., Beijing, China, 99.99%), Tris(2,2′- bipyridine) dichlororuthenium(II) hexahydrate (Ru(bipy)_3_Cl_2_, Aladdin Holdings Group Co., Ltd., Beijing, China, 99.95%), acetonitrile (MeCN, Adamas Pharmaceuticals, Inc., Shanghai, China, 99.9%), triethanolamine (TEOA, Aladdin Holdings Group Co., Ltd., Beijing, China), cobalt chloride hexahydrate (CoCl_2_·6H_2_O, Alfa Aesar A Johnson Matthey Company, Heysham, UK, 98%), 2,2′-bipyridine (2,2′-bipy, Alfa Aesar A Johnson Matthey Company, Heysham, UK, 98%), 3,3′-bipyridine (3, 3′-bipy, Aladdin Holdings Group Co., Ltd., Beijing, China, 97%), 2,4-bipyridine (2,4-bipy, Tokyo Chemical Industry, Tokyo, Japan, 98%), 4,4′-bipyridine (4,4′-bipy, Aladdin Holdings Group Co., Ltd., Beijing, China, 97%), 4,4′-dimethyl-2,2′-bipyridine (4,4′-Me_2_-bipy, Adamas Pharmaceuticals, Inc., Shanghai, China, 98%), 5,5′-dimethyl-2,2′-bipyridine (5,5′-Dm2-bipy, Adamas Pharmaceuticals, Inc., Shanghai, China, 98%), 6,6′-dimethyl-2,2′-bipyridine (6,6′-Me_2_-bipy, Adamas Pharmaceuticals, Inc., Shanghai, China, 98%), 4,4′-ditert butyl-2,2′-bipyridine (4,4′-tBu2-bipy, Adamas Pharmaceuticals, Inc., Shanghai, China, 98%), and 4,4′-dioctyl-2,2′-bipyridine (4,4′-Non2-bipy, Adamas Pharmaceuticals, Inc., Shanghai, China, 98%). Ultrapure water (resistivity ~18 MΩ·cm) was used throughout the experiments.

### 3.2. Crystallographic Studies

Single-crystal X-ray diffraction data for several metal complexes (Co(bipy)_3_Cl_2_, Co(2,3-bipy)_3_Cl_2_, Co(4,4′-Me_2_-2,2′-bipy)_3_Cl_2_, Co(4,4′-tBu-2,2′-bipy)_3_Cl_2_, Co(4,4′-bipy)_3_Cl_2_) were obtained by employing a Bruker D8 QUEST diffractometer (Bruker, Billerica, MA, USA) equipped with Mo-Kα radiation (λ = 0.71073 Å) in ω scan mode.

### 3.3. UV–Vis Test

The UV–vis absorption was measured on an UV–vis spectrophotometer (Varian Cary 500, Palo Alto, CA, USA). The samples were prepared as the reaction medium (V_MeCN_:V_H2O_:V_TEOA_ = 4:1:1), and the CoCl_2_ and organic ligands (nCo:nL = 1:3) were directly mixed before measurement.

### 3.4. FT-IR Inspection

Fourier-transformed infrared (FT-IR) spectra were recorded using a Nicolet Magna 670 (Thermo Fisher Scientific, Waltham, MA, USA) FT-IR spectrometer and the samples were mixed with KBr at a concentration of ca. 1 wt. %. The CoCl_2_ and various ligands (n_Co_:n_L_ = 1:3) were added directly into the mortar and then mixed with KBr.

### 3.5. Electrochemical Measurements

Electrochemical measurements were performed with a CHI 660E (Shanghai Chenhua, Shanghai, China) workstation in a conventional three-electrode cell, using a Pt plate as the counter electrode and saturated calomel electrode (SCE) as the reference electrode. The photoelectrodes were prepared by a typical coating method: the aqueous slurries of CdS were quantitatively coated on ITO glass substrates. The films on the ITO glass substrate were dried in air and annealed at 100 °C for 1 h as the final photoelectrodes. The electrolyte solution (ca. 60 mL), saturated with N_2_ or CO_2_ and stirred with a magnetic stirrer, contained the same ingredients as the photocatalytic reduction system in each solution. The cell was then sealed and, at the same time, the three electrodes were immersed into the electrolyte because they were fixed on the cover of the cell. The electrolysis cell was placed into a constant temperature water bath of 20 °C. The electrocatalytic behavior was tested from +1 to −2 V using cyclic voltammetry.

### 3.6. Photocatalytic CO_2_ Reduction Experiments

All experiments were carried out in a Schlenk flask (80 mL) under an atmospheric pressure of CO_2_ (1 atm). In the Schlenk flask, CdS (50 mg), 10 μmol CoCl_2_·6H_2_O accompanied with 30 μmol organic ligands were added in the mixtures (4 mL MeCN, 1 mL H_2_O and 1 mL TEOA). MeCN and TEOA were used as the solvent and sacrificial agent, respectively, while water provides the hydrogen source during the reaction. The system was vacuum-degassed and refilled with pure CO_2_ gas. This process was repeated three times and after the last cycle the flask was backfilled with CO_2_. Then, the system was irradiated with two counter non-focused 50 W white LED light source (λ > 420 nm) under vigorous stirring at 15 °C, controlled by a water-cooling system. The produced gases (CO, H_2_) were detected by a gas chromatography (Agilent 7890, Agilent Technologies, Santa Clara, CA, USA) equipped with a packed column (TDX-1 mesh 42/10) and Ar as the carrier gas.

## 4. Conclusions

In summary, a series of Co(bipy)_3_Cl_2_ molecular photocatalysts with varied alkyl substituents were systematically evaluated to clarify the effects of substituent type and coordination position on photocatalytic CO_2_ reduction. Substitution at the 4-position on bipyridine with methyl, tert-butyl, and nonyl groups led to decreased catalytic activity by 13.2%, 29.6%, and 98%, respectively, while a 5-position methyl group caused a 71.1% reduction. Ligands such as 6,6′-Me_2_-bipy, 2,4-bipy, and 3,3′-bipy exhibited no observable CO_2_ conversion under identical conditions, indicating the critical influence of both steric hindrance and ligand geometry on coordination. Spectroscopic and electrochemical analyses confirmed the formation and stability of coordination complexes, as well as their relationship with photocatalytic efficiency. Overall, these findings demonstrate that photocatalytic CO_2_ reduction by tris(bipyridine)cobalt(II) complexes is clearly hindered by bulky ligands, primarily due to steric effects that interfere with metal–ligand coordination and the formation of catalytically active species. These results underscore the strong dependence of catalytic performance on ligand structure and offer a molecular-level rationale for the design of improved photocatalytic systems for CO_2_ conversion.

## Figures and Tables

**Table 1:** Study of photocatalytic performances for various ligands ^[a]^.

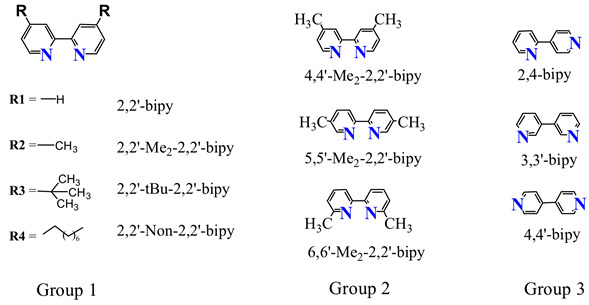					
**Entry**	**Ligands**	**CO/μmol**	**H_2_/μmol**	**CO + H_2_/μmol**	**Sel./% ^[b]^**
1	CdS	1.2	5.6	6.8	17.6
2	Co(II)+2,2′-bipy	/ ^[c]^	0.1	/	/
3	CdS+2,2′-bipy	0.1	6.5	6.6	1.5
4	Ru(bipy)_3_Cl_2_	49.8	10.1	59.9	83.1
5	Dark	/	/	/	/
6	2,2′-bipy	40.8	8.6	49.4	82.6
7	4,4′-Me_2_-2,2′-bipy	35.4	7.5	42.9	82.5
8	4,4′-tBu_2_-2,2′-bipy	28.7	6.2	34.9	82.2
9	4,4′-Non_2_-2,2′-bipy	0.8	1.5	2.3	0.34
10	5,5′-Me_2_-2,2′-bipy	11.8	3.6	15.4	76.6
11	6,6′-Me_2_-2,2′-bipy	/	3.2	3.2	/
12	2,4-bipy	/	2.8	2.8	/
13	3,3′-bipy	/	0.5	0.5	/
14	4,4′-bipy	6.3	4.5	10.9	57.7

^[a]^ Reaction carried out at 20 °C and 2 h duration. ^[b]^ Selectivity = n_CO_/n_(CO+H2)_ × 100. ^[c]^ Not detected.

## Data Availability

No new data were created or analyzed in this study. Data sharing is not applicable to this article.

## Supplementary Materials

The following supporting information can be downloaded at: https://www.mdpi.com/article/10.3390/molecules30122573/s1, Table S1. Crystallographic data. Table S2. Comparison table of photocatalytic CO_2_ reduction systems. Figure S1. FT-IR result for complexes of CoCl_2_, 2, 2′-bipy and Co(2, 2′-bipy)_3_Cl_2_ (Inset: photos for 2, 2′-bipy and Co(2, 2′-bipy)_3_Cl_2_); Figure S2. FT-IR result for complexes of CoCl_2_, 4, 4′-Me_2_-2, 2′-bipy and Co(4, 4′-Me_2_-2, 2′-bipy)_3_Cl_2_ (Inset: photos for 4, 4′-Me_2_-2, 2′-bipy and Co(4, 4′-Me_2_-2, 2′-bipy)_3_Cl_2_); Figure S3. FT-IR result for complexes of CoCl_2_, 4, 4′-tBu_2_-2, 2′-bipy and Co(4, 4′-tBu_2_-2, 2′-bipy)_3_Cl_2_ (Inset: photos for 4, 4′-tBu_2_-2, 2′-bipy and Co(4, 4′-tBu_2_-2, 2′-bipy)_3_Cl_2_); Figure S4. FT-IR result for complexes of CoCl_2_, 4, 4′-Non_2_-2, 2′-bipy and Co(4, 4′-Non_2_-2, 2′-bipy)_3_Cl_2_ (Inset: photos for 4, 4′-Non_2_-2, 2′-bipy and Co(4, 4′-Non_2_-2, 2′-bipy)_3_Cl_2_); Figure S5. FT-IR result for complexes of CoCl_2_, 5, 5’-Me_2_-2, 2’-bipy and Co(5, 5’-Me_2_-2, 2’-bipy)_3_Cl_2_; Figure S6. FT-IR result for complexes of CoCl_2_, 6, 6’-Me_2_-2, 2’-bipy and Co(6, 6’-Me_2_-2, 2’-bipy)_3_Cl_2_; Figure S7. FT-IR result for complexes of CoCl_2_, 2, 4-bipy and Co(2, 4-bipy)_3_Cl_2_; Figure S8. FT-IR result for complexes of CoCl_2_, 3, 3’-bipy and Co(3, 3’-bipy)_3_Cl_2_; Figure S9. FT-IR result for complexes of CoCl_2_, 4, 4’-bipy and Co(4, 4’-bipy)_3_Cl_2_; Figure S10. Cyclic voltammograms of the reduction wave of Co-species in the electrolyte with 2, 2-bipy after N_2_ and CO_2_ saturation; Figure S11. Cyclic voltammograms of the reduction wave of Co-species in the electrolyte with 4, 4’-Me_2_-2, 2-bipy after N_2_ and CO_2_ saturation; Figure S12. Cyclic voltammograms of the reduction wave of Co-species in the electrolyte with 4, 4’-tBu_2_-2, 2-bipy after N_2_ and CO_2_ saturation; Figure S13. Cyclic voltammograms of the reduction wave of Co-species in the electrolyte with 4, 4′-Non_2_-2, 2-bipy after N_2_ and CO_2_ saturation; Figure S14. Cyclic voltammograms of the reduction wave of Co-species in the electrolyte with 5, 5′-Me_2_-2, 2-bipy after N_2_ and CO_2_ saturation; Figure S15. Cyclic voltammograms of the reduction wave of Co-species in the electrolyte with 6, 6′-Me_2_-2, 2-bipy after N_2_ and CO_2_ saturation; Figure S16. Cyclic voltammograms of the reduction wave of Co-species in the electrolyte with 2, 4-bipy after N_2_ and CO_2_ saturation; Figure S17. Cyclic voltammograms of the reduction wave of Co-species in the electrolyte with 3, 3′-bipy after N_2_ and CO_2_ saturation; Figure S18. Cyclic voltammograms of the reduction wave of Co-species in the electrolyte with 4, 4′-bipy after N_2_ and CO_2_ saturation; Figure S19. Photos of reaction mediums after 30 min light irradiation; Figure S20. XRD pattern of the CdS sample.
