# Supplementary material for: Bulky Ligand-Induced Hindrance in Photocatalytic CO2 Reduction over Various Tris(bipyridine)cobalt(II) Chloride Complexes
Source: Molecules. 2025 Jun 13;30(12):2573. doi: 10.3390/molecules30122573 (PMC12196081; doi:10.3390/molecules30122573)
Supplement: Supplementary file 1 [file molecules-30-02573-s001.zip › molecules-3417708-supplementary.pdf]

Supplementary Information

Bulky Ligand-Induced Hindrance in Photocatalytic CO_2 Reduction over Various Tris(Bipyridine)Cobalt(II) Chloride Complexes

Jinliang Lin [1,*], Rongying Liao [1], Li Li [1], Shuli Yao [1], Shengkai Li [1], Yun Zheng [2] and Fei Fei [3]

[1] School of Intelligent Manufacturing and Materials Engineering, Gannan University of Science and Technology, Ganzhou 341000, China; liaorongying@gnust.edu.cn (R.L.); 9320080302@gnust.edu.cn (L.L.); 9320220065@gnust.edu.cn (S.Y.); 9320230036@gnust.edu.cn (S.L.)

[2] Fujian Provincial Key Laboratory of Biomass Low-Carbon Conversion, Huaqiao University, Xiamen 361021, China; zheng-yun@hqu.edu.cn

[3] Department of Chemical and Engineering, Zunyi Normal University, Zunyi 563000, China; feifei90092@163.com

* Correspondence: jinliang_lin@163.com; Tel.:+86-797-8312718

Table S1. Crystallographic Data.

Name	Co(bipy)$_3$Cl$_2$	Co(4, 4'-Me$_2$-2, 2'-bipy)$_3$Cl$_2$	Co(4, 4'-tBu-2, 2'-bipy)$_3$Cl$_2$	Co(2,3-bipy)$_3$Cl$_2$	Co(4, 4'-bipy)$_3$Cl$_2$
formula	$C_{30}H_{24}N_6CoCl_2$	$C_{32}H_{28}N_6CoCl_2$	$C_{38}H_{40}N_6CoCl_2$	$C_{30}H_{24}N_6CoCl_2$	$C_{30}H_{24}N_6CoCl_2$
formula weight	598.48	682.64	763.89	598.48	598.48
crystal system	triclinic	triclinic	triclinic	triclinic	triclinic
space group	$P\bar{1}$	$P\bar{1}$	$P\bar{1}$	$P\bar{1}$	$P\bar{1}$
a/Å	12.14	9.10	12.73	7.51	8.96
b/Å	12.16	11.35	17.50	10.67	9.91
c/Å	22.20	13.91	17.62	11.96	21.85
α/deg	90.33	67.91	73.83	89.88	89.74
β/deg	90.12	71.09	88.27	89.97	86.91
γ/deg	119.92	79.21	73.83	89.95	89.95
radiation	Mo-Kα λ=0.71073 Å	Mo-Kα λ=0.71073 Å	Mo-Kα λ=0.71073 Å	Mo-Kα λ=0.71073 Å	Mo-Kα λ=0.71073 Å

Note : Data are collected via Difference Vectors method

Table S2. Comparison Table of Photocatalytic CO_2 Reduction Systems.

Catalyst System	Light Source (λ)	Product	CO Yield (μ mol)	Selectivity (%)	Reference
Co(2,2'-bipy)$_3$Cl$_2$	>420 nm (LED)	CO, H_2	40.8	82.6	This work
Co(4,4'-Me$_2$-2,2'-bipy)$_3$Cl$_2$	>420 nm (LED)	CO, H_2	35.4	82.5	This work
Co(4,4'-tBu$_2$-2,2'-bipy)$_3$Cl$_2$	>420 nm (LED)	CO, H_2	28.7	82.2	This work
Ru(bipy)$_3$Cl$_2$	>420 nm (LED)	CO, H_2	49.8	83.1	Ref. [12]
Ni(qpy)	Solar	CO	23.5	90	Ref. [17]
Mn(bipy)(CO)$_3$Br	>400 nm	CO	12.4	78.5	Ref. [19]

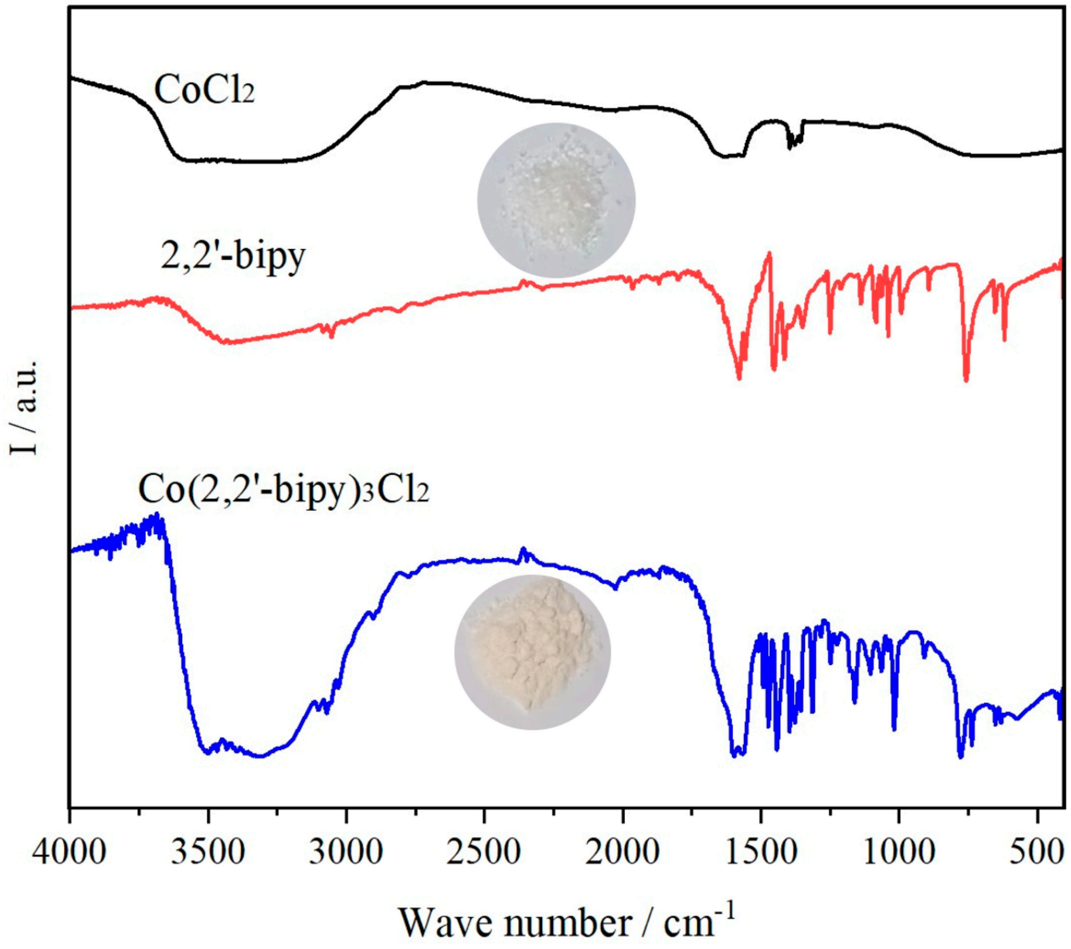

Figure S1 FT-IR result for complexes of $CoCl_2$, 2, 2’-bipy and Co(2, 2’-bipy)$_3$$Cl_2$

(Inset: photos for 2, 2’-bipy and Co(2, 2’-bipy)$_3$$Cl_2$).

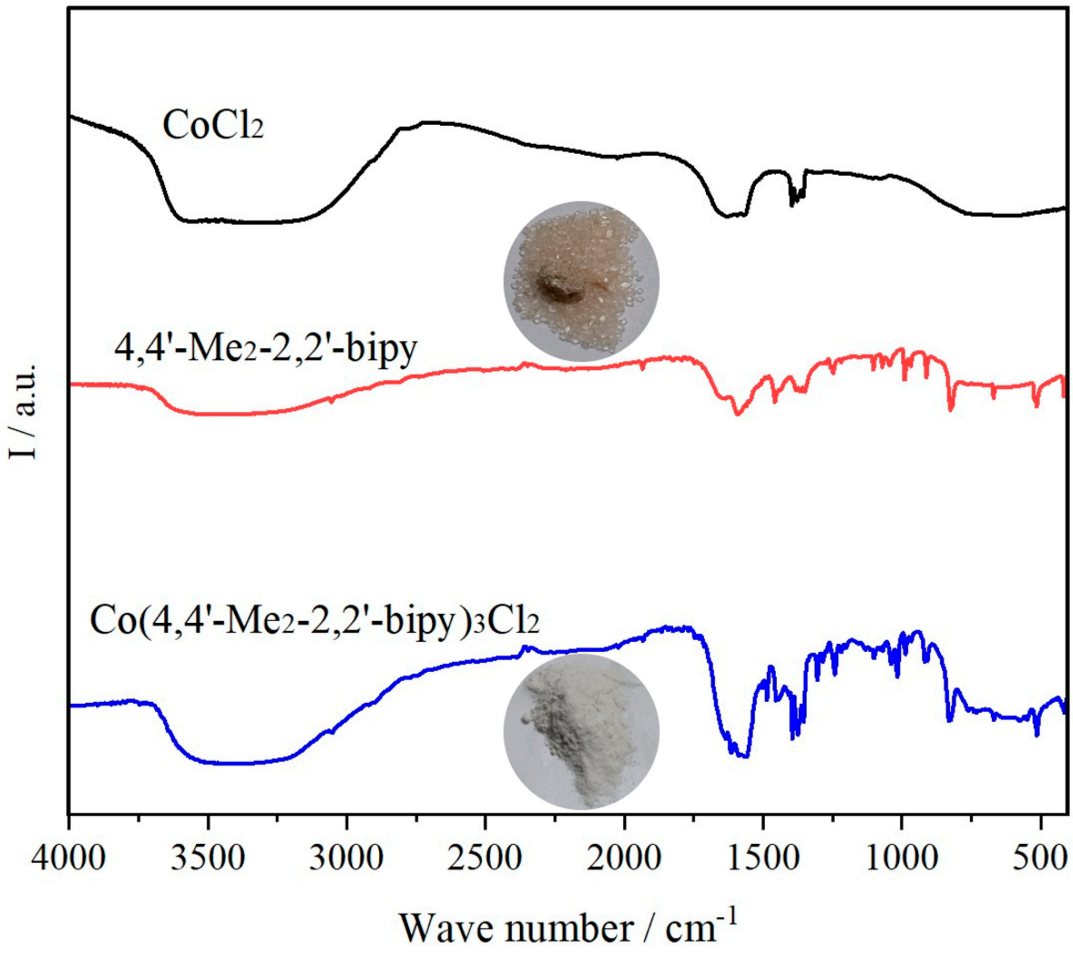

Figure S2 FT-IR result for complexes of $CoCl_2$, 4, 4'-Me_2-2, 2'-bipy and Co(4, 4'-Me_2-2, 2'-bipy)$_3Cl_2$ (Inset: photos for 4, 4'-Me_2-2, 2'-bipy and Co(4, 4'-Me_2-2, 2'-bipy)$_3Cl_2$).

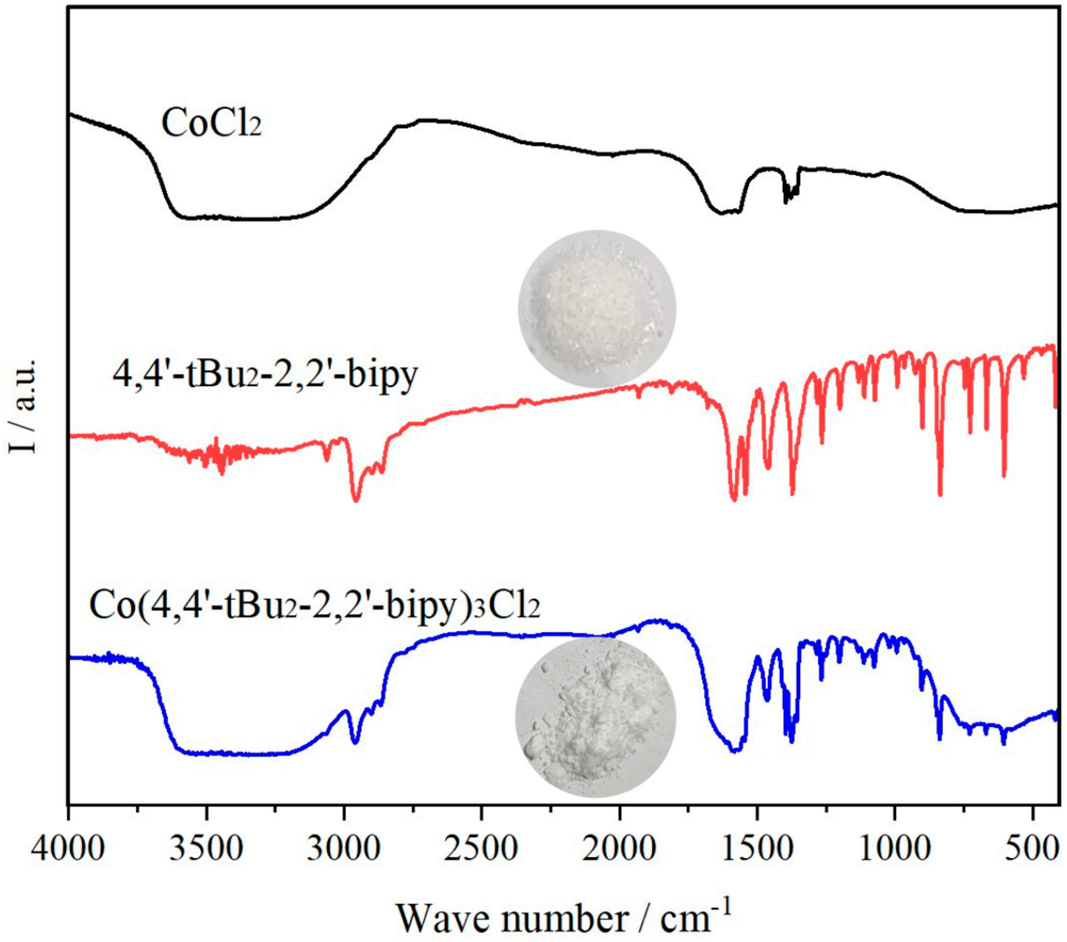

Figure S3 FT-IR result for complexes of $CoCl_2$, 4, 4'-tBu$_2$-2, 2'-bipy and Co(4, 4'-tBu$_2$-2, 2'-bipy)$_3$Cl$_2$ (Inset: photos for 4, 4'-tBu$_2$-2, 2'-bipy and Co(4, 4'-tBu$_2$-2, 2'-bipy)$_3$Cl$_2$).

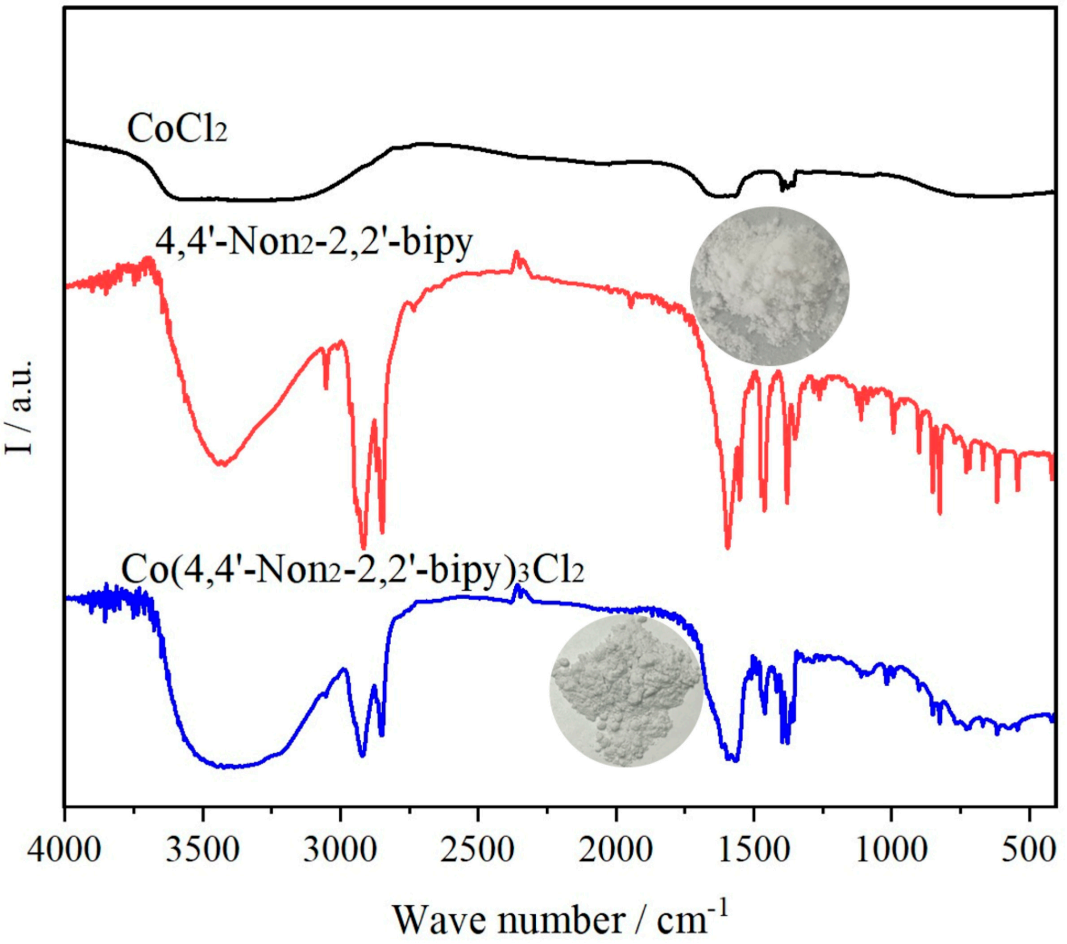

Figure S4 FT-IR result for complexes of $CoCl_2$, 4, 4'-Non_2-2, 2'-bipy and Co(4, 4'-Non_2-2, 2'-bipy)$_3Cl_2$ (Inset: photos for 4, 4'-Non_2-2, 2'-bipy and Co(4, 4'-Non_2-2, 2'-bipy)$_3Cl_2$).

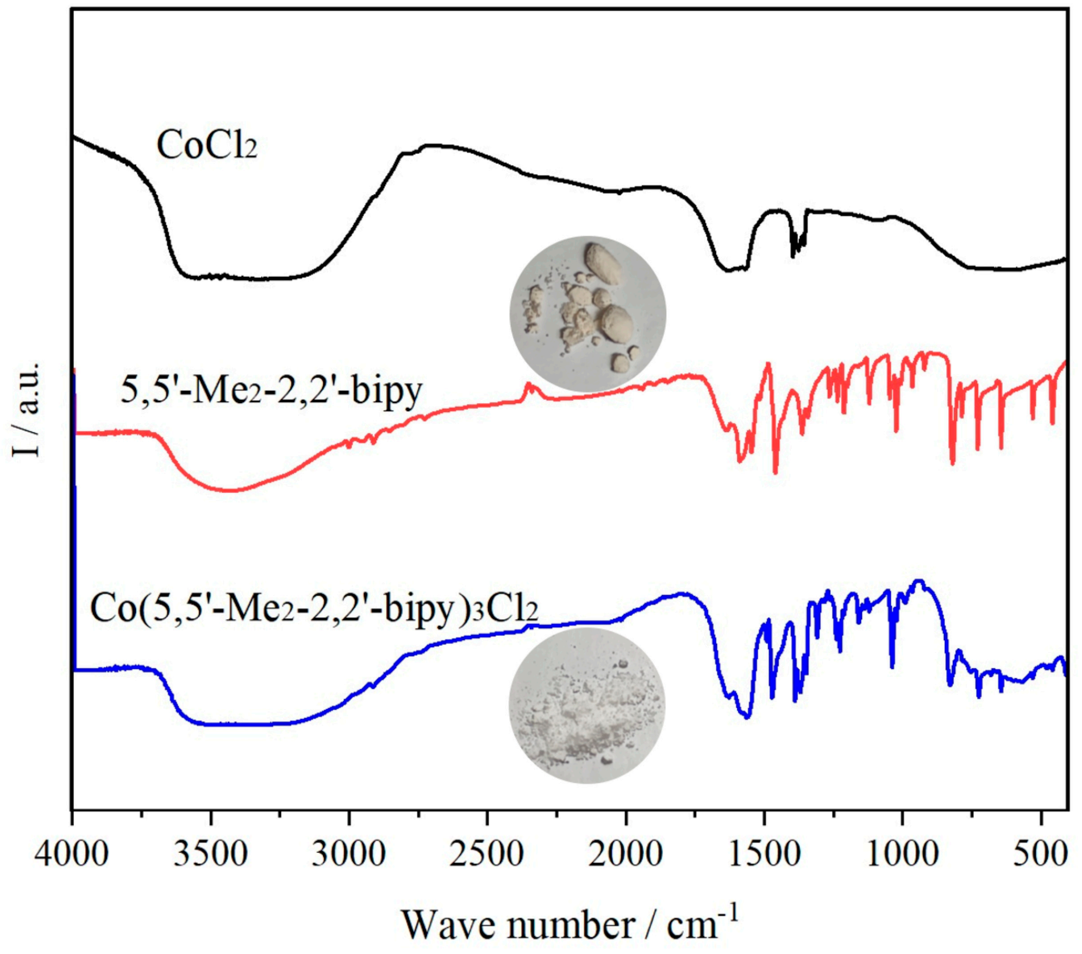

Figure S5 FT-IR result for complexes of $CoCl_2$, 5, 5'-Me_2-2, 2'-bipy and Co(5, 5'-Me_2-2, 2'-bipy)$_3Cl_2$ (Inset: photos for 5, 5'-Me_2-2, 2'-bipy and Co(5, 5'-Me_2-2, 2'-bipy)$_3Cl_2$).

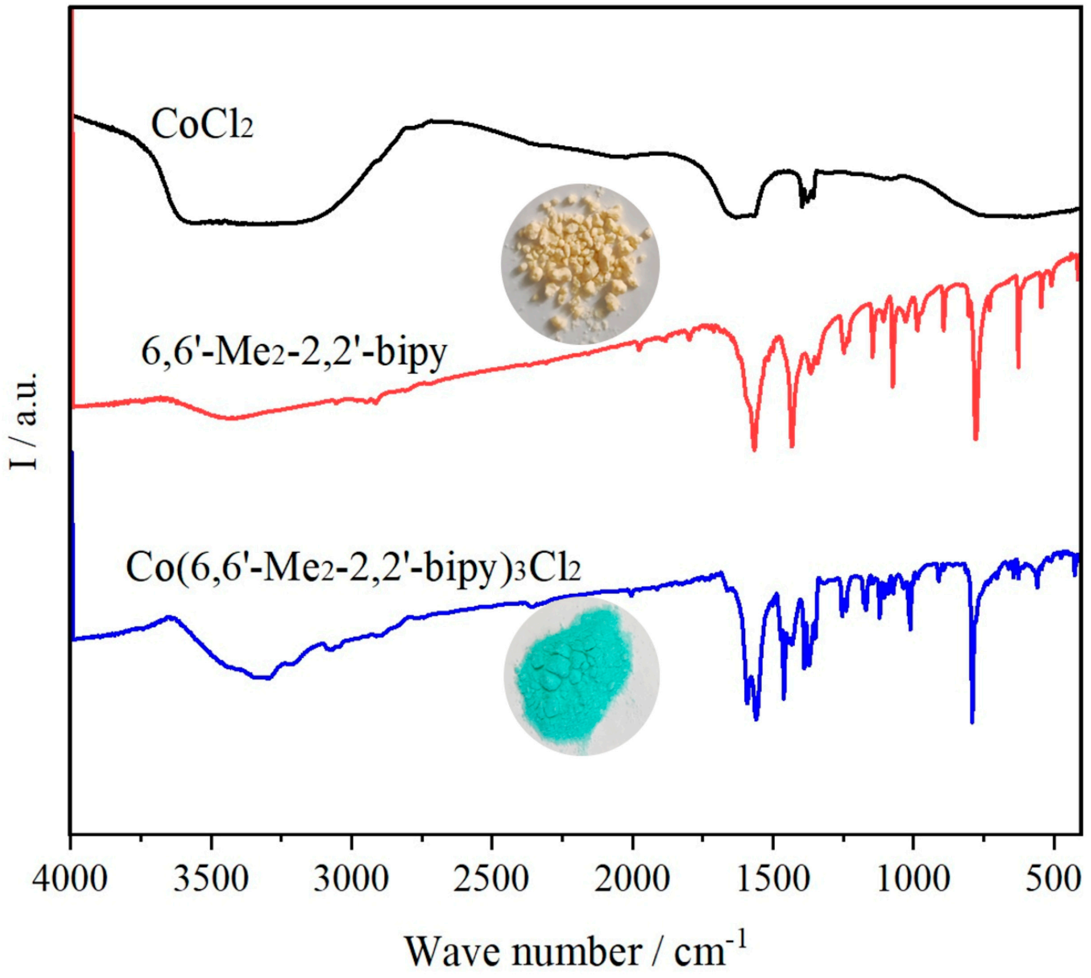

Figure S6 FT-IR result for complexes of $CoCl_2$, 6, 6'-Me_2-2, 2'-bipy and Co(6, 6'-Me_2-2, 2'-bipy)$_3Cl_2$ (Inset: photos for 6, 6'-Me_2-2, 2'-bipy and Co(6, 6'-Me_2-2, 2'-bipy)$_3Cl_2$).

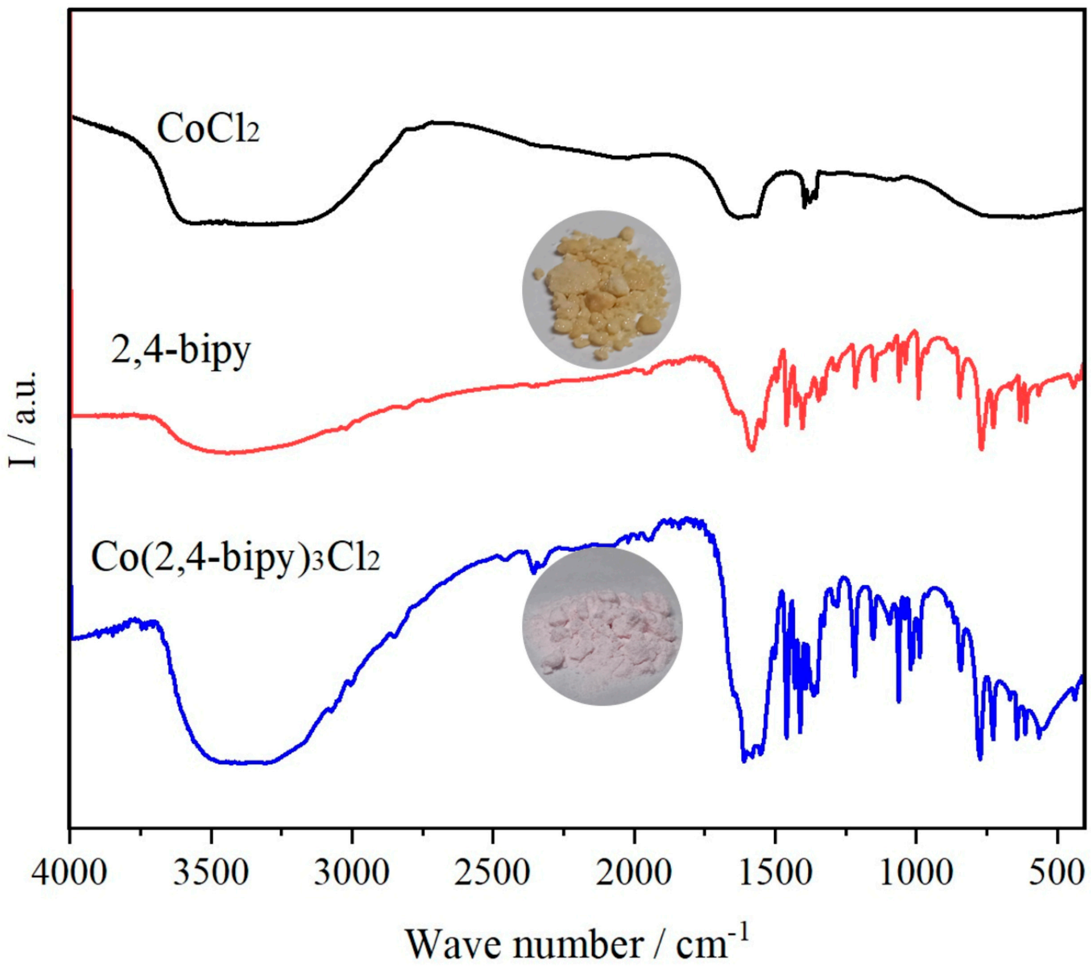

Figure S7 FT-IR result for complexes of $CoCl_2$, 2, 4-bipy and Co(2, 4-bipy)$_3$Cl$_2$

(Inset: photos for 2, 4-bipy and Co(2, 4-bipy)$_3$Cl$_2$).

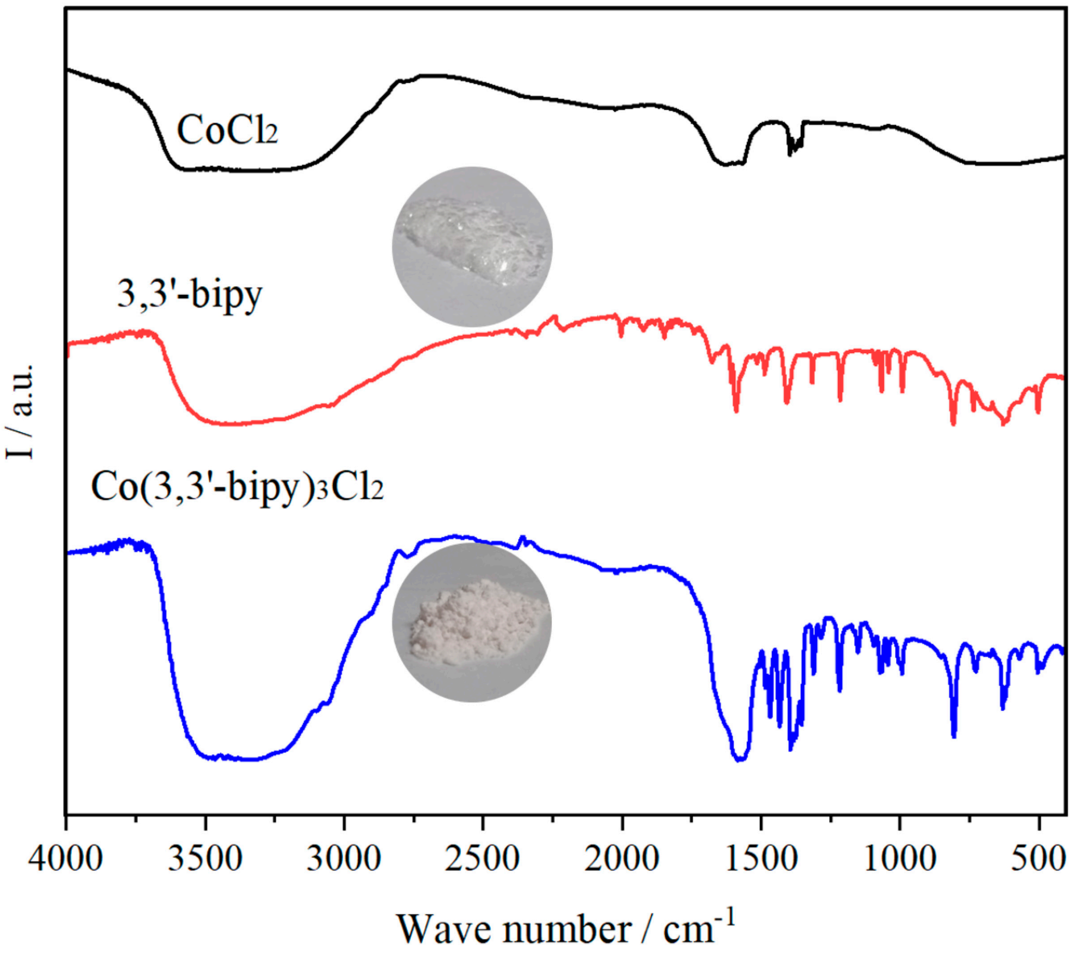

Figure S8 FT-IR result for complexes of $CoCl_2$, 3, 3'-bipy and Co(3, 3'-bipy)$_3Cl_2$ (Inset: photos for 3, 3'-bipy and Co(3, 3'-bipy)$_3Cl_2$).

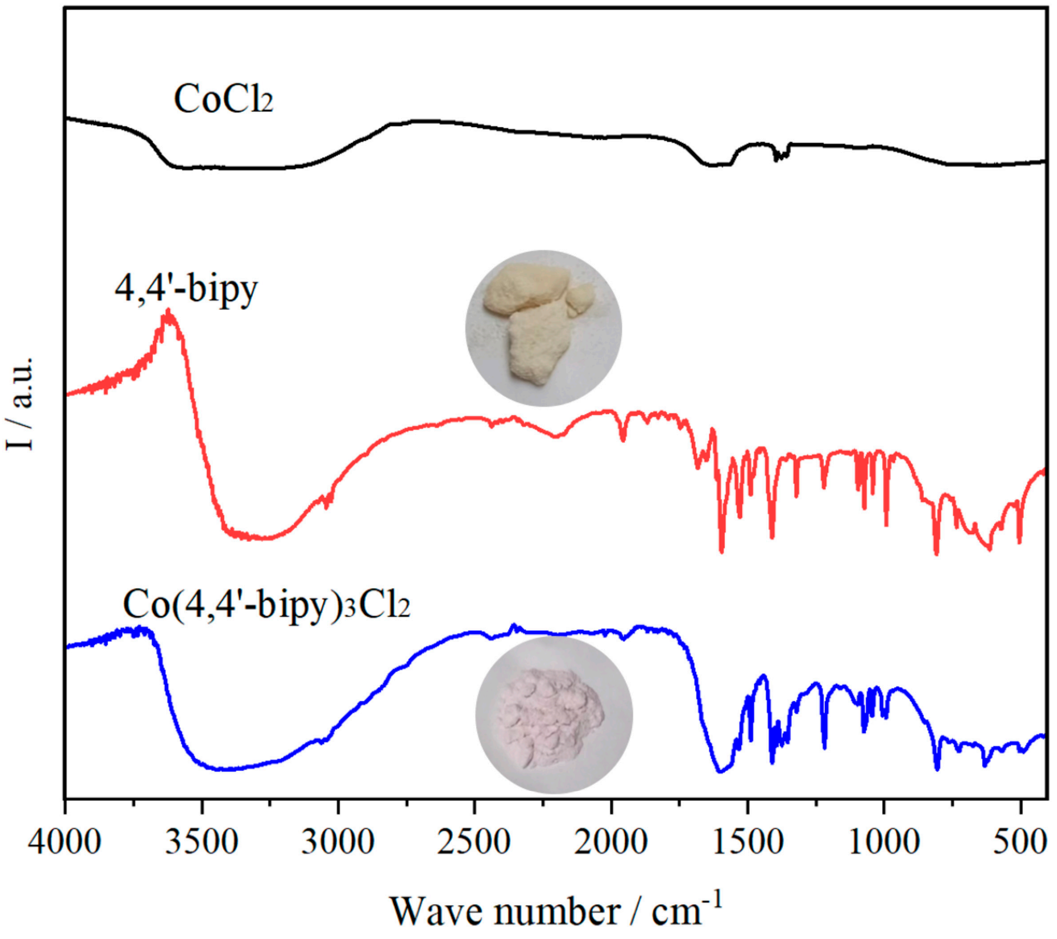

Figure S9 FT-IR result for complexes of $CoCl_2$, 4, 4'-bipy and Co(4, 4'-bipy)$_3$$Cl_2$

(Inset: photos for 4, 4'-bipy and Co(4, 4'-bipy)$_3$$Cl_2$).

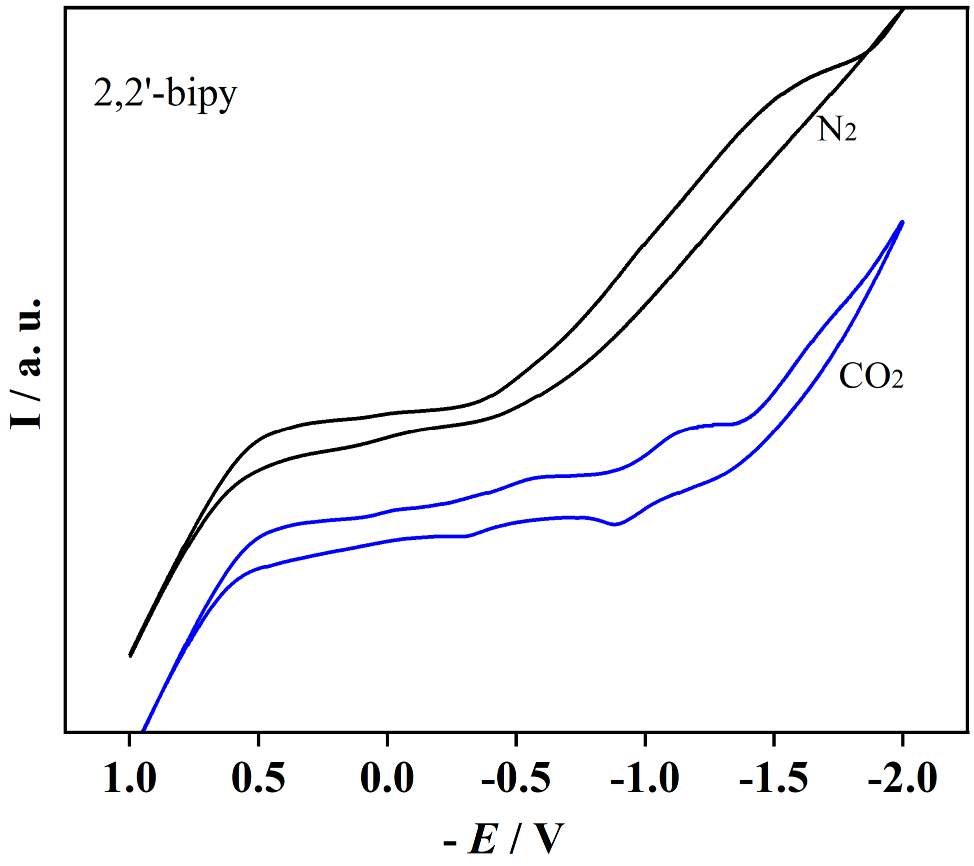

Figure S10 Cyclic voltammograms of the reduction wave of Co-species in the electrolyte with 2, 2-bipy after N_2 and CO_2 saturation.

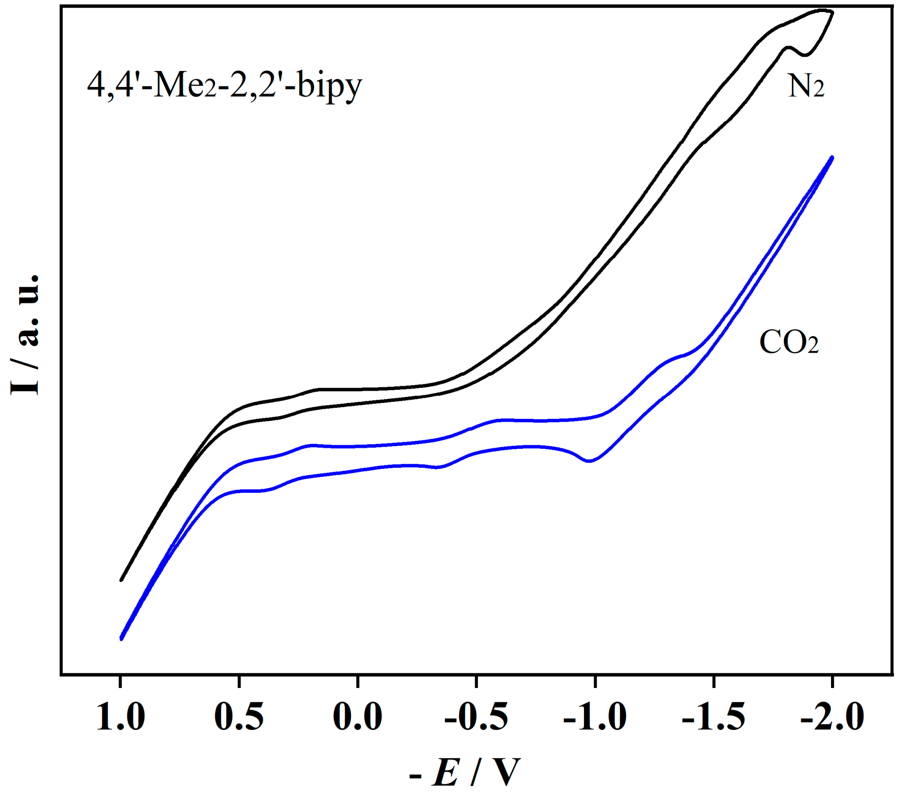

Figure S11 Cyclic voltammograms of the reduction wave of Co-species in the electrolyte with 4, 4'-Me_2-2, 2-bipy after N_2 and CO_2 saturation.

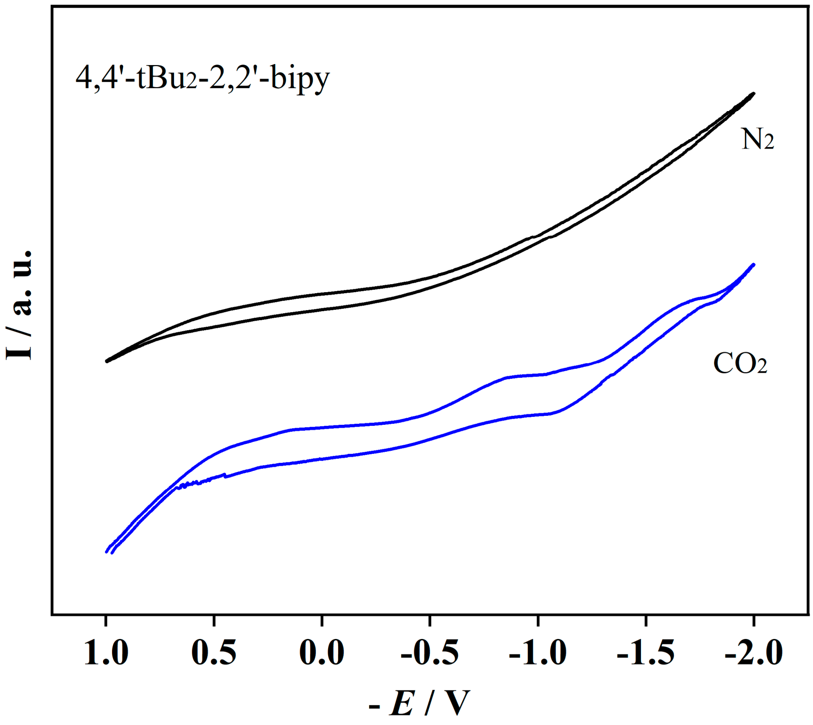

Figure S12 Cyclic voltammograms of the reduction wave of Co-species in the electrolyte with 4, 4’-tBu$_2$-2, 2-bipy after N_2 and CO_2 saturation.

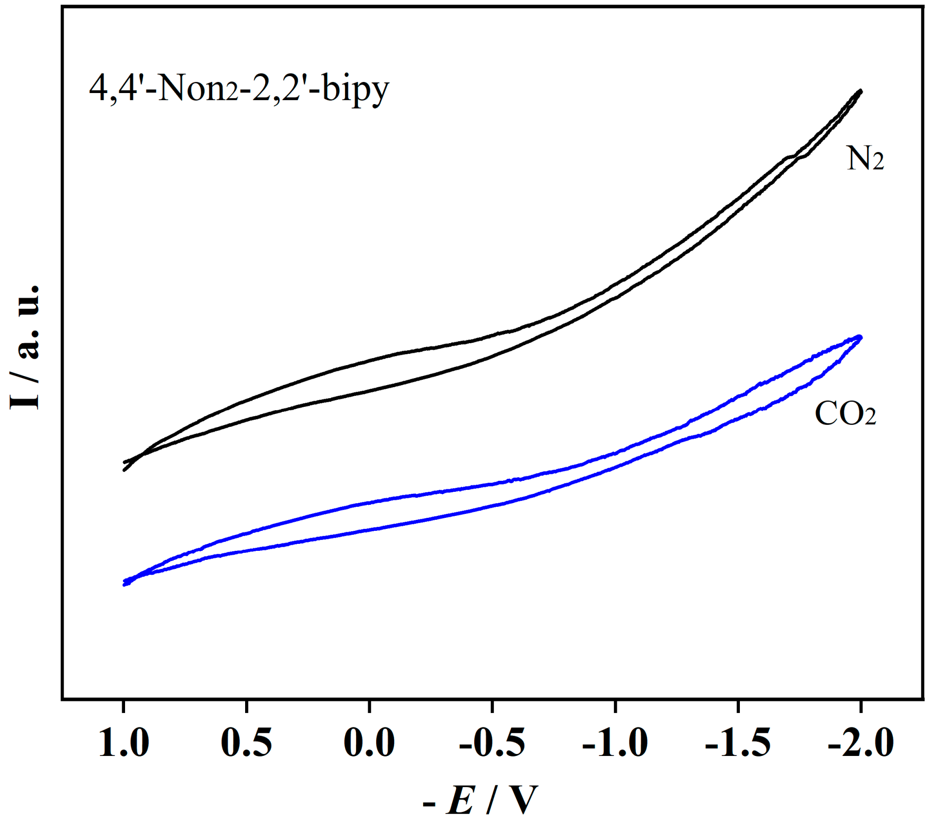

Figure S13 Cyclic voltammograms of the reduction wave of Co-species in the electrolyte with 4, 4'-Non2-2, 2-bipy after N_2 and CO_2 saturation.

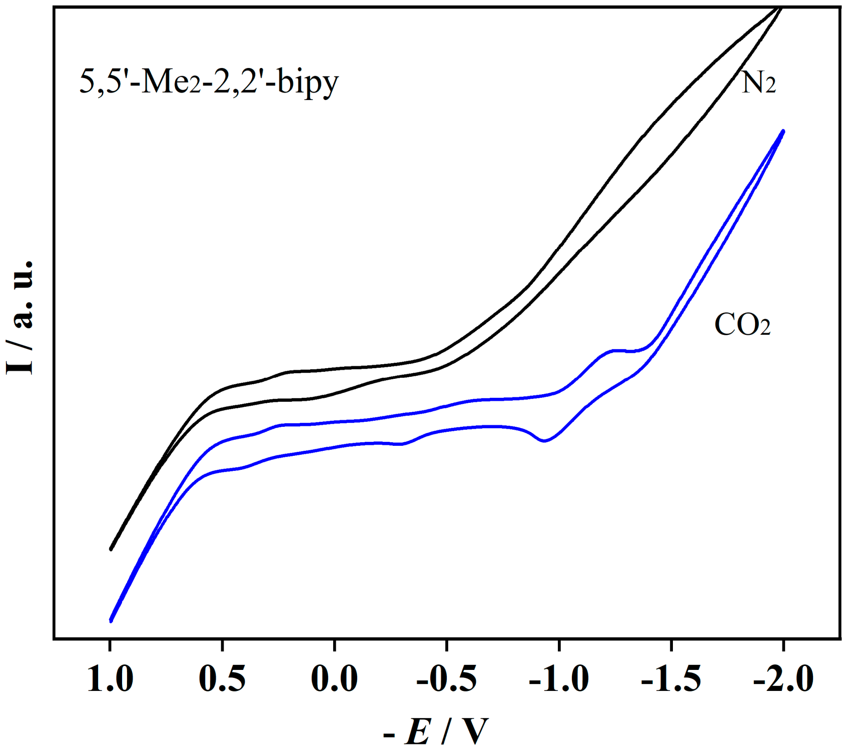

Figure S14 Cyclic voltammograms of the reduction wave of Co-species in the electrolyte with 5, 5'-Me_2-2, 2-bipy after N_2 and CO_2 saturation.

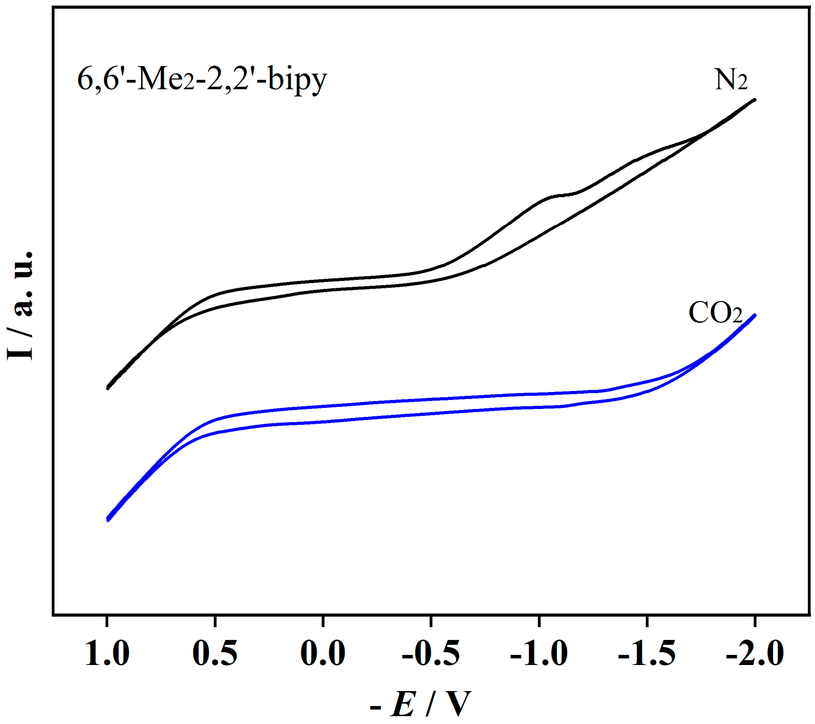

Figure S15 Cyclic voltammograms of the reduction wave of Co-species in the electrolyte with 6, 6’-Me$_2$-2, 2-bipy after N_2 and CO_2 saturation.

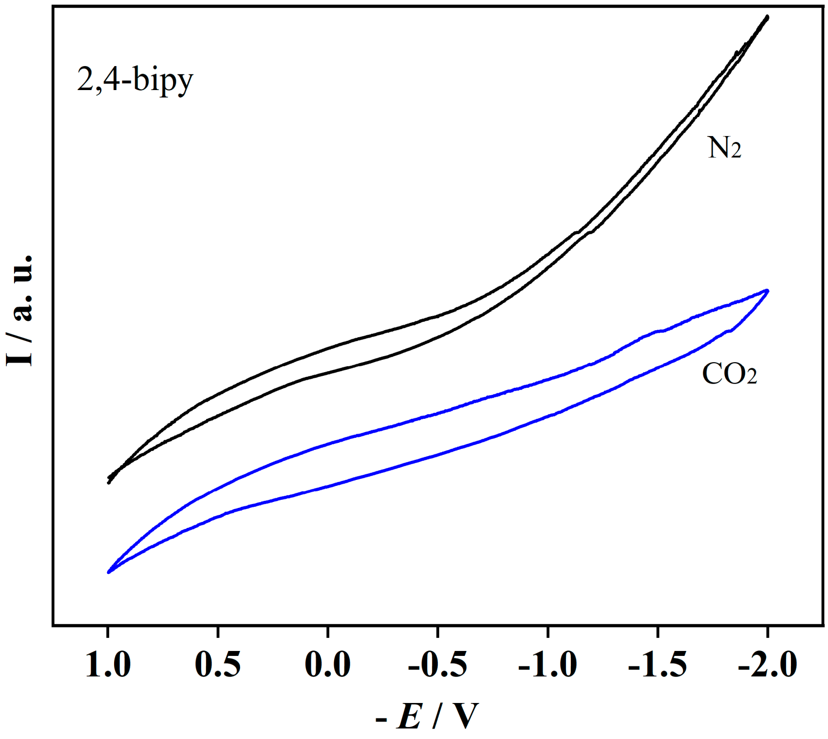

Figure S16 Cyclic voltammograms of the reduction wave of Co-species in the electrolyte with 2, 4-bipy after N_2 and CO_2 saturation.

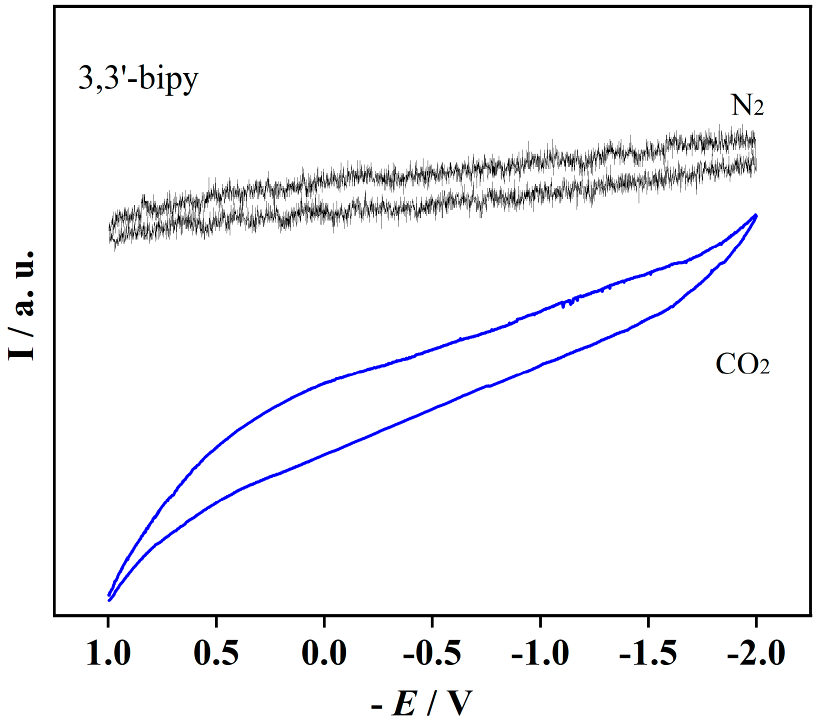

Figure S17 Cyclic voltammograms of the reduction wave of Co-species in the electrolyte with 3, 3’-bipy after N_2 and CO_2 saturation.

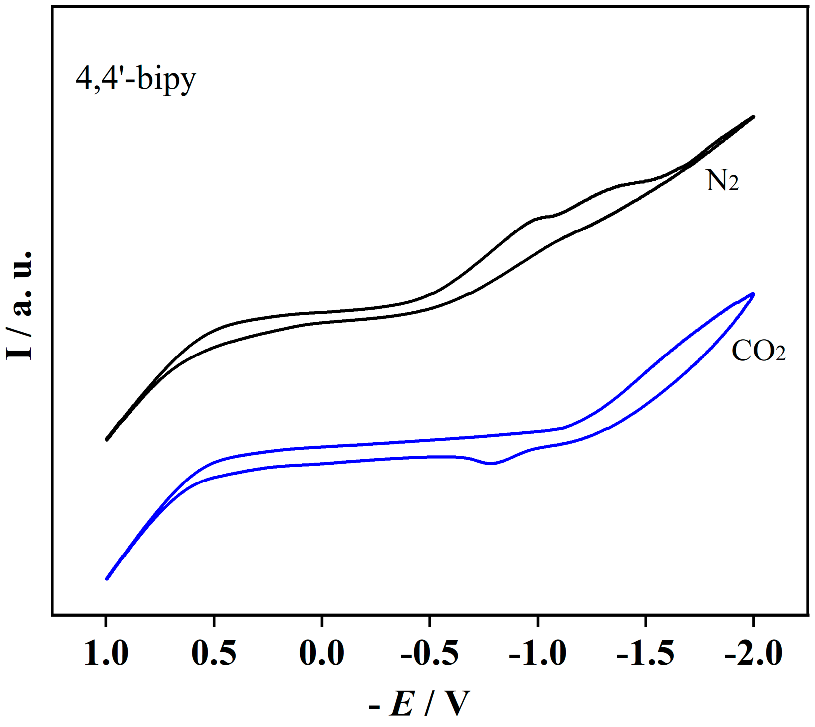

Figure S18 Cyclic voltammograms of the reduction wave of Co-species in the electrolyte with 4, 4'-bipy after N_2 and CO_2 saturation.

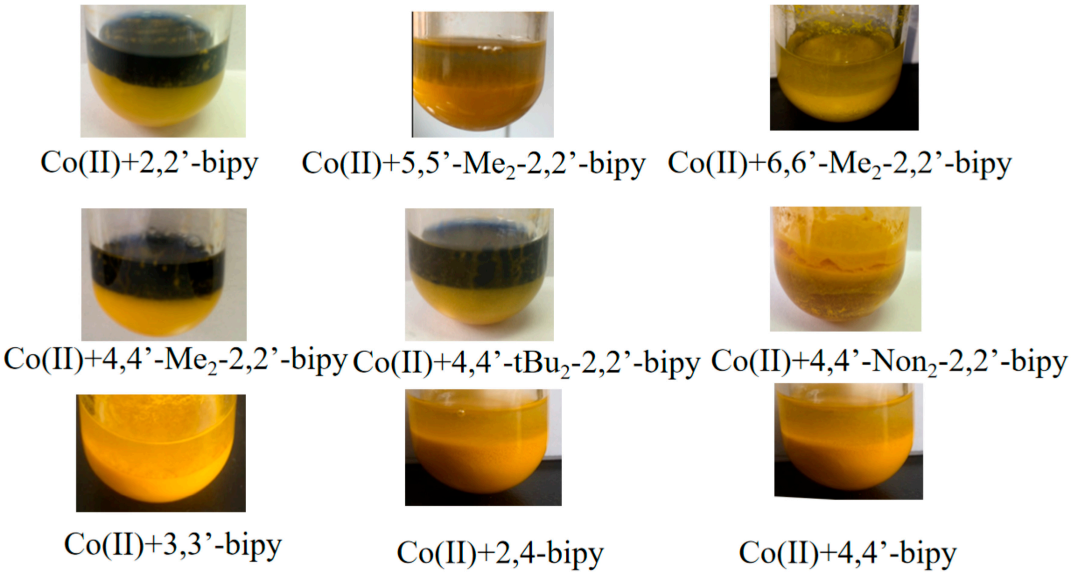

Figure S19 Photos of reaction mediums after 30 min light irradiation.

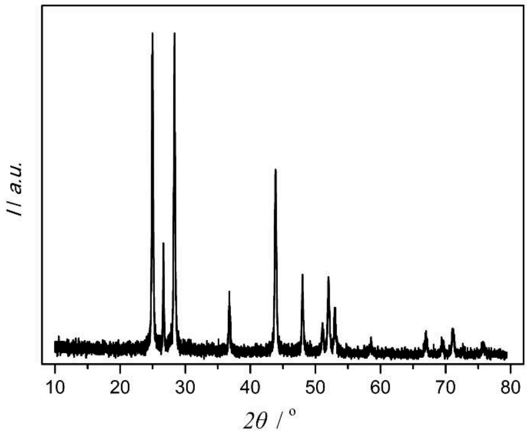

Figure S20 XRD pattern of the CdS sample.

.
